# Severe Diltiazem Poisoning Managed With CytoSorb Hemoadsorption and Supportive Therapies: A Case Report

**DOI:** 10.1097/CCE.0000000000001454

**Published:** 2026-08-03

**Authors:** Juul M. Cox, Julia A. M. Verwaaijen, Ruben M. F. Hendriks, Dorien Smeets, Suzanne van Rijswijk, Tessa M. Bosch

**Affiliations:** 1 Department of Hospital Pharmacy, Maasstad Hospital, Rotterdam, The Netherlands.; 2 Department of Clinical Pharmacology and Toxicology MaasstadLab, Maasstad Hospital, Rotterdam, The Netherlands.; 3 Department of Intensive Care Medicine, Maasstad Hospital, Rotterdam, The Netherlands.

**Keywords:** calcium channel blocker poisoning, diltiazem, hemoadsorption, hemoperfusion, renal replacement therapy, toxicokinetics

## Abstract

**BACKGROUND:**

Severe intoxication with nondihydropyridine calcium channel blockers, such as diltiazem, is associated with high morbidity and mortality. Evidence supporting extracorporeal treatment strategies remains limited. This case integrates serial toxicokinetic measurements with adjunctive hemoadsorption in sustained-release diltiazem poisoning.

**CASE SUMMARY:**

A 73-year-old man presented with coma, complete atrioventricular block, refractory hypotension, and severe lactic acidosis. Despite optimized supportive therapy including vasopressors, calcium supplementation, hyperinsulinemic-euglycemic therapy, and continuous renal replacement therapy, he remained hemodynamically unstable. CytoSorb hemoadsorption was initiated 6 hours after ICU admission. Shortly thereafter, sinus rhythm was restored, serum lactate rapidly declined, and vasopressor and insulin requirements were progressively reduced, allowing discontinuation of extracorporeal support. The patient recovered and was discharged home.

**CONCLUSIONS:**

Retrospective analysis demonstrated elevated diltiazem concentrations with delayed, capacity-limited elimination. This case supports considering hemoadsorption in unstable patients with severe diltiazem poisoning despite conventional measures and highlights the need for systematic pharmacokinetic evaluation in future studies.

**KEY POINTS**

**Question**: Can CytoSorb hemoadsorption serve as an effective adjunctive therapy in severe sustained-release diltiazem poisoning with refractory shock despite optimized conventional treatment?

**Findings**: In this single-patient case report, initiation of CytoSorb hemoadsorption during continuous renal replacement therapy was temporally associated with rapid hemodynamic stabilization, lactate normalization, restoration of sinus rhythm, and progressive discontinuation of vasopressors and hyperinsulinemic-euglycemic therapy.

**Meaning**: Hemoadsorption may be a clinically relevant adjunct in selected cases of life-threatening calcium channel blocker poisoning and warrants systematic pharmacokinetic evaluation in future studies.

Diltiazem, a nondihydropyridine calcium channel blocker (CCB), is prescribed for angina and mild to moderate hypertension. Overdose with CCBs, particularly diltiazem and verapamil, may cause profound bradycardia, hypotension, and conduction disturbances, which carry high mortality (1, 2). Severe CCB poisoning is managed with IV fluids, vasopressors, calcium supplementation, hyperinsulinemic-euglycemic therapy (HIET), and IV lipid emulsion in refractory cases (1, 2).

Although diltiazem has a low molecular weight (415 Da), its large volume of distribution (Vd, ~5.3 L/kg), lipophilicity (log *p* of approximately 2.7–3.0), and extensive protein binding (80%) limit dialyzability. Evidence supporting extracorporeal treatments remains limited (3), and the Extracorporeal Treatments in Poisoning does not recommend it for diltiazem poisoning.

This report describes a life-threatening sustained-release (SR) diltiazem intoxication in which adjunctive CytoSorb hemoadsorption led to rapid hemodynamic and biochemical improvement. The case demonstrates how hemoadsorption may eliminate excess unbound diltiazem and its metabolites, potentially preventing escalation to extracorporeal membrane oxygenation (ECMO) and mitigating irreversible organ damage. This observation adds to previous reports of severe diltiazem intoxication in which patients deteriorated despite maximal conventional support and improved only after ECMO (4).

## CASE DESCRIPTION

The patient provided written informed consent for publication of this case report, including clinical details and figures. A 73-year-old man with a history of bipolar disorder, hypertension, type 2 diabetes mellitus, ischemic heart disease, chronic kidney disease, and peripheral artery disease with chronic leg ulcers presented to the emergency department with bradypnea (8/min), hypoxemia (peripheral oxygen saturation 85%), hypotension (65/35 mm Hg), and bradycardia (50 beats/min). Electrocardiography revealed complete atrioventricular block. His Glasgow Coma Scale score was 3 and serum glucose 15.5 mmol/L. Home medication included diltiazem and tramadol. SR diltiazem ingestion was suspected and later confirmed by toxicokinetic analysis.

Initial management targeted presumed septic shock due to infected leg ulcers, complicated by acute kidney injury and liver failure, with suspected drug toxicity as a contributing factor. The patient received naloxone, isoprenaline, and empirical antibiotics. He regained consciousness and was transferred to the ICU.

A preexisting do-not-resuscitate and do-not-intubate order was respected. High-flow nasal oxygen and vasopressors (starting dose norepinephrine 0.6 µg/kg/min and vasopressin 0.03 international units [IUs]/min) were started. Isoprenaline was replaced by dobutamine due to persistent bradycardia and moderately reduced left ventricular function on bedside ultrasound. Suspected CCB intoxication prompted initiation of HIET (0.5 IU/kg/hr) and IV calcium gluconate (50 mg/kg/hr). Gastrointestinal decontamination was withheld as accumulation of CCB rather than intentional overdose was initially suspected.

**Figure 1.**
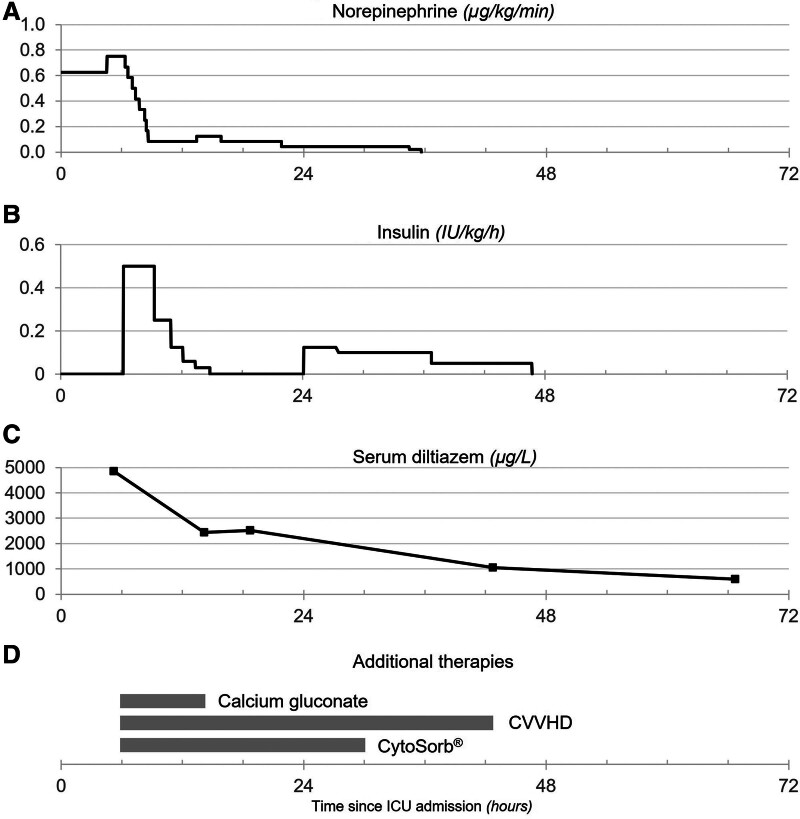
Temporal relationship between hemodynamic support, metabolic recovery, and plasma diltiazem concentrations following CytoSorb initiation. All panels share the same *x*-axis (time since ICU admission [hr]). **A**, Norepinephrine dose (µg/kg/min) decreased rapidly following CytoSorb initiation. **B**, Insulin infusion rate during hyperinsulinemic-euglycemic therapy (international units [IUs]/kg/hr) was progressively reduced. **C**, Plasma diltiazem concentrations (µg/L) decreased following CytoSorb initiation, with a more gradual decline thereafter. **D**, Duration of additional supportive therapies: CytoSorb hemoadsorption, continuous venovenous hemodialysis (CVVHD), and IV calcium gluconate. The concurrent reductions in vasopressor and inotrope requirements, insulin dose, and serum lactate, alongside declining plasma diltiazem concentrations, suggest a close temporal association between the initiation of CytoSorb hemoadsorption and clinical stabilization.

Six hours after admission, metabolic acidosis and increased work of breathing persisted despite sodium bicarbonate administration, prompting initiation of continuous renal replacement therapy (CRRT). A femoral double-lumen catheter was placed. Continuous venovenous hemodialysis (CVVHD) with regional citrate anticoagulation was initiated on a multiFiltratePRO system equipped with an Ultraflux AV1000S hemofilter using trisodium citrate (Cifoban), calcium-free dialysate (Ci-Ca Dialysate K4 Plus), and calcium chloride (Caddera; all Fresenius Medical Care, Bad Homburg, Germany). CytoSorb hemoadsorption (CytoSorbents Corporation, Princeton, NJ) was added pre-filter and maintained from 6 to 30 hours after admission (**Fig. 1**). Blood and dialysate flow rates were 160 mL/min and 3200 mL/hr, respectively, yielding a prescribed CRRT dose of 40 mL/kg/hr.

Over the next 24 hours, sinus rhythm was restored and lactate levels declined, allowing tapering of vasopressors and insulin therapy. CytoSorb hemoadsorption was discontinued after 24 hours, after which both CRRT and vasopressors were successfully weaned (Fig. 1). On ICU day 3, the patient disclosed intentional diltiazem ingestion. By day 5, he was transferred to a general medical ward and discharged home after 18 days of hospitalization.

Retrospective toxicological analysis confirmed severe diltiazem intoxication. Serial plasma concentrations are presented in **Table 1**: 4847 µg/L at 5.2 hours, 2435 µg/L at 14.2 hours, 2525 µg/L at 18.7 hours, 1059 µg/L at 42.7 hours, and 594 µg/L at 66.7 hours after admission. This pattern indicated sustained absorption of a SR formulation and delayed, capacity-limited elimination. No other cardioactive or toxic agents were detected.

**TABLE 1.** Observed Plasma Diltiazem Concentrations and Estimated Elimination Rates

Time Since Admission (hr)	Diltiazem Concentration (µg/L)	Natural Logarithm of Concentration	Change in the Natural Logarithm of Concentration Over Time (per hr)	Apparent Half-Life (hr)
5.2	4847	1.578	N/A	N/A
14.2	2435	0.890	–0.076	9.1
18.7	2525	0.926	+0.008	N/A
42.7	1059	0.057	–0.036	19.1
66.7	594	–0.521	–0.024	28.8

N/A = not applicable.

For each time interval, the apparent elimination rate constant (*k*) was approximated as change in the natural logarithm of concentration over time (Δln(C)/Δt). Under first-order elimination, *k* = –slope, and the half-life (t½) equals ln(2)/*k*. For each interval, an *k* was approximated as Δln(C)/Δt, and an apparent t½ as ln(2)/k. These values are descriptive only: in the early intervals, ongoing absorption from the sustained-release formulation overlaps with elimination, so the calculated half-lives do not represent true first-order elimination and should not be interpreted as a defined kinetic transition. Positive slope values reflect measurement variability or ongoing absorption/redistribution rather than elimination. Concentrations at 14.2 and 18.7 hr exhibit a slight positive slope, which may indicate measurement variability or minor reabsorption or redistribution rather than elimination.

## DISCUSSION

This case illustrates the toxicokinetic complexity of SR diltiazem overdose and highlights the potential role of CytoSorb hemoadsorption when conventional measures are insufficient. Diltiazem is highly lipophilic, widely distributed (Vd ≈ 5.3 L/kg), and ~80% protein-bound. Hepatic cytochrome P450 3A4 metabolism yields active metabolites, while renal elimination is negligible (< 5% urine excretion).

Diltiazem has a half-life of 4–8 hours. Serial diltiazem concentrations (Table 1 and **Fig. S1*F***, https://links.lww.com/CCX/B662) showed a measured peak followed by a progressive prolongation of the apparent half-life, likely reflecting ongoing SR absorption and saturation of hepatic metabolism rather than true first-order elimination. This should be interpreted descriptively rather than as evidence of a defined kinetic transition. Using a Vd of 5 L/kg for a 75 kg individual, the peak implies an in-body load of approximately 1.8 g and a minimum ingested dose of greater than or equal to 4–5 g (assuming 40–50% bioavailability); these are rough approximations dependent on assumed pharmacokinetic parameters and an uncertain peak concentration. The prolonged toxicity was probably driven by sustained SR gastrointestinal absorption together with saturation of hepatic metabolism reducing the first-pass effect. This, possibly in combination with shock-related impaired hepatic function, might have contributed to an extended elimination half-life. Further studies should clarify the kinetics of adsorber saturation and guide optimal replacement intervals during hemoadsorption therapy (5).

After initiation of CVVHD and CytoSorb hemoadsorption, lactate declined, sinus rhythm was restored, and vasopressors were weaned (Fig. 1; see also **Fig. S1** [https://links.lww.com/CCX/B662] for lactate and full vasopressor data). Although causality cannot be established, the temporal association suggests enhanced removal of unbound diltiazem. Plasma concentrations remained relatively stable between 14.2 and 18.7 hours, possibly indicating adsorber saturation, although this cannot be confirmed without pre- and post-adsorber measurements. Causality cannot be established from a single, uncontrolled case, as HIET, calcium supplementation, vasopressor support, and CVVHD are important confounders. The temporal association between CytoSorb initiation and clinical improvement after failure of optimized therapy should therefore be regarded as hypothesis-generating rather than as evidence of a causal effect. No adverse events or device-related complications occurred, highlighting the safety of this approach.

Hypoalbuminemia (~20 g/L; **Table 2**) likely increased the unbound fraction of diltiazem, amplifying toxicity but also facilitating extracorporeal removal, as only unbound drug is adsorbable. These observations align with the proposed mechanism of action: hemoadsorption is most effective in a high-concentration, high-free-fraction range. Together with published reports describing successful CytoSorb elimination in verapamil (6) and amlodipine intoxications (4), our findings strengthen the emerging rationale for adsorption-based therapy in severe CCB overdose. At our center, CytoSorb is reserved for life-threatening intoxications involving small, lipophilic agents with large distribution volumes (e.g., log *p* > 1; molecular weight < 60 kDa) when standard measures fail, in line with institutional guidance.

**TABLE 2.** Laboratory Findings at Admission

Test Items	Value at Admission	Laboratory Reference	Units
Blood count			
Leukocytes	15.5	4.0–10.0	× 10^9^
Hemoglobin	8.7	7.5–10.0	mmol/L
Hematocrit	0.43	0.35–0.45	L/L
Thrombocytes	307	150–400	× 10^9^
Arterial blood gas			
pH	7.18	7.35–7.45	
Pco_2_	34	42–50	mm Hg
Base excess	–15	–3 to 3	
Bicarbonate	13	23–27	mmol/L
Electrolytes			
Sodium	139	135–145	mmol/L
Potassium	4.1	3.5–5.0	mmol/L
Chloride	111	97–107	mmol/L
Calcium (total)	2.43	2.20–2.65	mmol/L
Calcium (ionized)	1.27	1.15–1.32	mmol/L
Metabolism			
C-reactive protein	32	< 10	mg/L
Creatinine	278	45–84	µmol/L
Blood urea nitrogen	16.3	2.4–6.4	mmol/L
Bilirubin, total	4	< 17	µmol/L
Gamma-glutamyltransferase	40	< 55	U/L
Alkaline phosphatase	62	< 115	U/L
Aspartate aminotransferase	29	< 35	U/L
Alanine aminotransferase	19	< 45	U/L
Lactate dehydrogenase	236	< 250	U/L
Lipase	43	13–60	U/L
Creatine kinase	65	< 171	U/L
Glucose	16.4	4.1–6.1	mmol/L
Lactate	7.9	0.5–2.2	mmol/L
Albumin	26	35–52	g/L

Comprehensive laboratory results obtained shortly after hospital admission, before initiation of CytoSorb therapy. The data indicate metabolic acidosis (pH 7.18, base excess –15 mmol/L, lactate 7.9 mmol/L), acute-on-chronic kidney disease (creatinine 278 µmol/L, blood urea nitrogen 16.3 mmol/L), hyperglycemia (16.4 mmol/L), and hypoalbuminemia (26 g/L), consistent with shock and multiple organ dysfunction. All reference ranges refer to local laboratory standards.

Comparable delayed or biphasic elimination patterns have been reported in severe CCB overdoses. A review of CCB toxicity found that SR diltiazem intoxications often required up to 22 hours of HIET for hemodynamic stabilization (7). In a mixed intoxication with 7200 mg diltiazem and a similar peak concentration of 4778 µg/L, hemodynamic improvement only occurred after starting HIET and lipid emulsion, 9 hours after ICU admission (8). In contrast, our patient stabilized rapidly following hemoadsorption and supportive therapies, suggesting that extracorporeal removal of unbound diltiazem may have accelerated recovery. In published series, hemodynamic stabilization typically requires prolonged HIET, often exceeding 24 hours (9, 10), suggesting that the early recovery in our case may reflect accelerated toxin clearance attributable to hemoadsorption.

This report has several limitations, including sparse sampling, limiting kinetic modeling. As there was no established protocol for CytoSorb initiation, pre- and post-adsorber samples were not obtained to quantify extraction efficiency. Consequently, the extraction ratio and apparent clearance during hemoadsorption could not be quantified, and an increase in systemic diltiazem clearance attributable to CytoSorb was not directly demonstrated. The estimated timing of adsorber saturation is inferred from the plasma concentration-time course and should be regarded as tentative. Furthermore, active metabolites, in particular desacetyl diltiazem, were not measured, as the retrospective toxicological analysis quantified only the parent compound. Because these metabolites retain appreciable pharmacologic activity, measurement of diltiazem alone may have underestimated the total active exposure, and the effect of hemoadsorption on metabolite concentrations could not be evaluated. Therefore, this report should be regarded as proof of concept rather than definitive evidence of efficacy. However, the combination of pharmacokinetic data, pathophysiological plausibility, and temporal correlation between CytoSorb initiation and clinical improvement provides a credible signal that warrants further study. Clinicians managing severe CCB poisoning should consider early hemoadsorption, collect paired pre- and post-adsorber samples when possible, and document pharmacokinetic and hemodynamic outcomes.

## CONCLUSIONS

In this case of severe diltiazem intoxication, the improvement in hemodynamic and metabolic parameters observed after initiation of CytoSorb hemoadsorption was temporally associated with its use and supports its potential as an adjunctive therapy when conventional measures fail. Hemoadsorption may remove the unbound toxic fraction of highly lipophilic or protein-bound drugs without adverse effects. Clinicians should monitor for adsorber saturation and consider timely adsorber replacement if ongoing treatment is needed. In our case, the plasma concentration-time course suggested possible adsorber saturation after approximately 8 hours, although this could not be confirmed without extraction data. Future studies should quantify extraction efficiency, characterize saturation kinetics, and define pharmacokinetic thresholds to optimize hemoadsorption in CCB and other lipophilic, protein-bound drug intoxications.

## Supplementary Material
