# Supplementary figures and images for: Severe Diltiazem Poisoning Managed With CytoSorb Hemoadsorption and Supportive Therapies: A Case Report
Source: Crit Care Explor. 2026 Aug 3;8(8):e1454. doi: 10.1097/CCE.0000000000001454 (PMC13433092; doi:10.1097/CCE.0000000000001454)

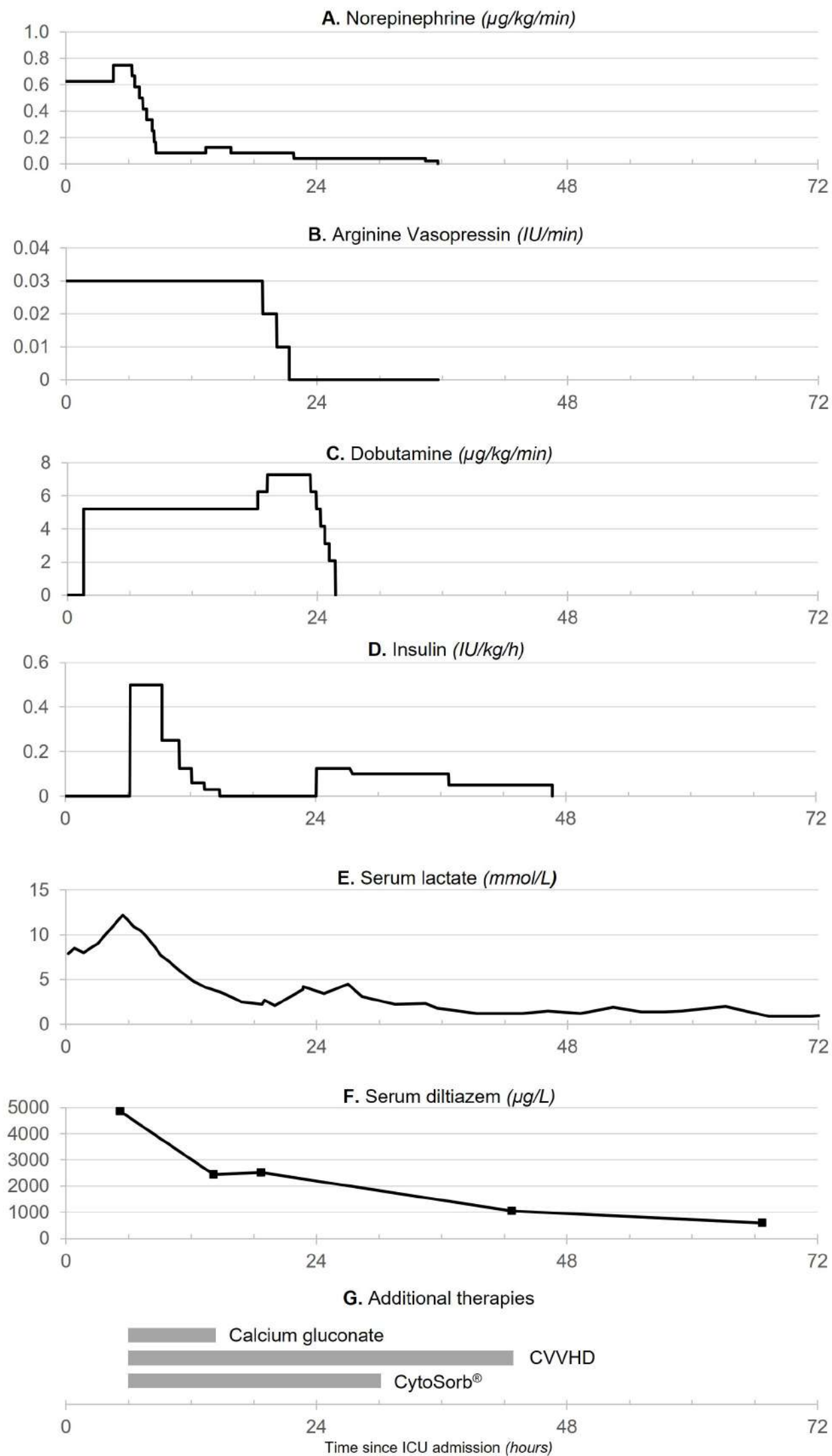

Supplement: Supplementary file 1 [file cc9-8-e1454-s001.pdf]
